# Mixed *Legionella pneumophila* Serogroup Infection in a Single Patient: Case Report and Literature Review

**DOI:** 10.3390/idr18050093

**Published:** 2026-08-26

**Authors:** Bram Vanmechelen, Fedoua Echahidi, Stijn Jonckheere, Patricia Vandecandelaere, Ines Malysse, Oriane Soetens, Charlotte Michel

**Affiliations:** 1 Department of Microbiology, Universitair Ziekenhuis Brussel (UZ Brussel), Vrije Universiteit Brussel (VUB), 1090 Brussels, Belgium; 2 Vitality Research Group, Vrije Universiteit Brussel (VUB), 1090 Brussels, Belgium; 3 Department of Laboratory Medicine, Jan Yperman Hospital, 8900 Ypres, Belgium; 4 Department of Internal Medicine, Jan Yperman Hospital, 8900 Ypres, Belgium

**Keywords:** *Legionella pneumophila*, Legionnaires’ disease, *Legionella pneumonia*, mixed Legionella infection, Legionella serogroup, Belgium

## Abstract

Background: Legionnaires’ disease (LD) is most often described as caused by a single *Legionella pneumophila* (Lp) serogroup (SG), typically SG1. However, mixed infections involving multiple SGs or even different *Legionella* species can occur within a single host. Such cases remain underrecognized and pose challenges for diagnosis, clinical management, and outbreak investigation. Case presentation: A 61-year-old woman developed acute fever, malaise, and productive cough after visiting a wellness spa in France, with radiological findings consistent with a lower respiratory tract infection. The urinary antigen test (UAT) for Lp was negative, but respiratory multiplex PCR was weakly positive. Subsequent testing at the Belgian National Reference Centre (NRC) for *Legionella* revealed an unexpected discordance between an in-house SG1-specific PCR and culture-based findings. Further analysis identified co-infection with two distinct Lp strains, SG1 (sequence type, ST146) and SG10 (ST1267), using a combination of direct sequence-based typing (SBT) and culture with serogrouping of multiple colonies. The patient improved rapidly after treatment with oral levofloxacin. Conclusions: Mixed Lp SG infections are likely underrecognized in routine practice, as neither UAT, commercial molecular assays, nor characterization of a single cultured isolate may adequately capture within-host strain diversity. Comprehensive diagnostic approaches combining culture with analysis of multiple colonies and molecular tests are essential for accurate case detection and source attribution. Referral to NRCs is recommended when advanced typing methods are unavailable. This first reported Belgian case illustrates the potential implications for patient management and epidemiological investigations, underscoring the need for broader diagnostic strategies.

## 1. Introduction

Legionnaires’ disease (LD) is a severe pneumonia caused by bacteria of the genus *Legionella*, most commonly *L. pneumophila* (Lp). These intracellular bacteria inhabit water and other moist environments, replicating within protozoa [1]. Sixteen serogroups (SGs) of Lp have been described, with SG1 accounting for the vast majority of human cases and representing the only target of most commercially available urinary antigen tests (UATs) [1,2]. LD presents with clinical features similar to those of other types of pneumonia, making microbiological testing essential for accurate diagnosis [3]. Historically, clinical and epidemiological investigations of LD have assumed that disease in any given patient results from infection with a single Lp strain. However, recent studies have demonstrated that patients may harbor multiple Lp strains or even different *Legionella* species simultaneously [4,5,6,7].

Water systems, including those in hospitals, hotels, and cruise ships, can harbor multiple *Legionella* strains simultaneously. These environments provide ideal conditions for biofilm formation and inter-strain exchange, raising the possibility of polymicrobial exposure during a single inhalation event [8,9,10,11,12]. These findings are clinically and epidemiologically significant, as they can complicate diagnostic interpretation, delay appropriate care, and obscure source attribution in outbreak settings.

The most recent European Centre for Disease Prevention and Control (ECDC) report (2023) indicates that the European Union/European Economic Area (EU/EEA) experienced the highest notification rate of LD to date, following an increasing trend observed prior to the COVID-19 pandemic (see Supplementary Table S1). In 2023, 79% of cases were diagnosed exclusively by UAT, whereas fewer than 10% were confirmed by culture. Mixed Lp SG infections accounted for less than 1% of culture-confirmed cases, a number that likely reflects diagnostic limitations rather than true rarity [13]. Reliance on a UAT-based diagnostic strategy may therefore contribute to systematic underrecognition of infections involving non-SG1 strains or other *Legionella* species [2]. Although PCR has substantially improved the sensitivity of *Legionella* detection and is increasingly used as a first-line diagnostic tool, mixed infections may still remain unrecognized, as commercial assays are designed to detect qualitatively the presence of *Legionella* rather than to characterize strain diversity and therefore do not distinguish between multiple co-infecting Lp SGs or strains [14].

Despite increasing molecular resolution in outbreak investigations, the true frequency of mixed Lp SG infections remains insufficiently defined. Although cases have been reported from Germany, Spain, and other countries, no such infection has yet been documented in Belgium [4]. We report, to our knowledge, the first Belgian case of mixed Lp SG infection confirmed by multi-colony culture analysis and PCR and review the literature to highlight its clinical and epidemiological significance.

## 2. Case Presentation

A 61-year-old woman presented to the emergency department of a regional hospital in West Flanders, Belgium, in March 2025, with acute onset of malaise, fever with chills and a productive cough with white sputum. At presentation, she was febrile (38.1 °C) but hemodynamically stable, with a blood pressure of 113/72 mmHg, heart rate of 74 beats/min, and oxygen saturation of 100% on room air and no signs of respiratory distress. Her symptoms had started the previous evening. She denied significant dyspnea and had no recent antibiotic exposure. The patient had returned a few days earlier from a holiday in the Vosges region of France, where she visited a wellness spa. The patient had a history of chronic inflammatory pulmonary disease, with organizing pneumonia confirmed by lung biopsy, and used inhaled corticosteroids for allergic airway disease.

Physical examination showed a productive cough, but clear breath sounds bilaterally. Laboratory evaluation demonstrated systemic inflammation, with elevated C-reactive protein (97.8 mg/L) and leukocytosis (10.9 × 10^3^/µL), while serum sodium levels, renal, and liver function parameters were within the normal range. No organ dysfunction was observed. Initial respiratory pathogen PCR testing on nasopharyngeal swab was negative for SARS-CoV-2, influenza, and respiratory syncytial virus. Chest CT revealed bilateral multifocal centrilobular nodules, tree-in-bud pattern, and consolidations, consistent with an infectious bronchopneumonic process. Therefore, an additional multiplex respiratory PCR panel was subsequently performed on the same sample, including detection of common atypical bacteria (*Bordetella pertussis*, *Chlamydia pneumoniae*, *Mycoplasma pneumoniae*, and Lp).

While the Lp UAT performed as standard-of-care was negative (TRU Legionella Test, Meridian Bioscience, Cincinnati, OH, USA), the multiplex respiratory PCR panel tested weakly positive for Lp (QIAstat-Dx Respiratory Panel, QIAGEN, Germany; see Supplementary Figure S1). To further investigate this weakly positive PCR result, a sputum sample was sent to the National Reference Centre (NRC) for *Legionella pneumophila* (Lp) (UZ Brussel, Brussels, Belgium) for confirmation of diagnosis and was tested using a specific in-house real-time quadruplex PCR, a combined protocol of two previously described *Legionella* real-time PCR methods [15,16]. The PCR consists of the simultaneous amplification of three *Legionella* targets, *wzm* (SG1-specific), *mip* (*Lp*-specific), 23S–5S (*Legionella* spp.) and, as an internal control, Herpes Virus-1 (PhHv-1) glycoprotein B target (samples are spiked with PhHv-1) [17]. The PCR was performed on the CFX96 Deepwell Dx real-time PCR system (BIO-RAD, Hercules, CA, USA). The sample was found positive for all three *Legionella* PCR targets (*wzm*, *mip* and 23S-5S). Based on PCR results alone, the presence of Lp SG1 could be confirmed; however, the subsequent culture of the respiratory sample at the NRC initially identified only a non-SG1 isolate, which contrasted with the SG1-specific PCR findings. To exclude the possibility of technical or analytical error, the molecular analysis was repeated, yielding concordant results and confirming the initial PCR findings. To resolve this discrepancy, multiple colonies from the same culture were further characterized, based on subtle differences in colony morphology, ultimately demonstrating the presence of two distinct Lp strains belonging to SG1 and SG2–15, respectively, with the SG2–15 strain being more frequently recovered (approximately 4 out of 6 colonies). Sequence-based typing (SBT) of the isolates later confirmed two distinct sequence types, ST146 (2,10,18,10,2,1,6) and ST1267 (2,6,48,6,48,5,40) [18,19,20,21,22].

**Figure 1:**
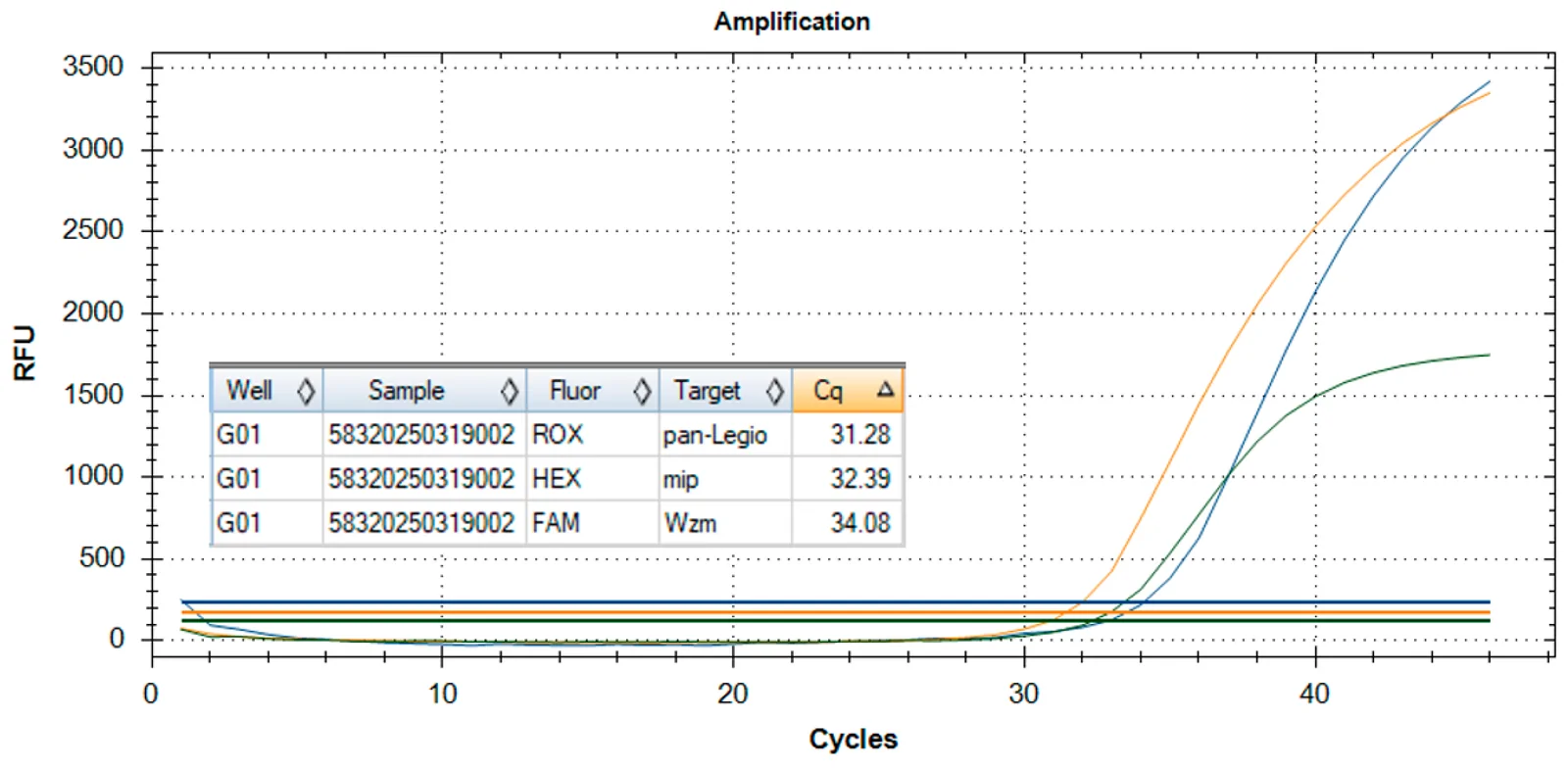
Real-time PCR amplification curves of *Legionella pneumophila* targets. The pan-Legio target (23-5S rRNA) is shown in orange, the *mip* target (*L. pneumophila*) in blue and the *wzm* SG1-specific target in green. The higher Cq value observed for the *wzm* target compared to the other targets could be consistent with a lower relative abundance of the SG1 strain in the sample.

Careful re-examination of the PCR curves revealed that the Cq value for the *wzm* SG1-specific target was approximately two cycles higher than for the *mip* (Lp) target, which suggested a lower relative amplification of the SG1-specific PCR target compared to the pneumophila-specific PCR target, which at the end confirmed our findings (Figure 1).

**Figure 2:**
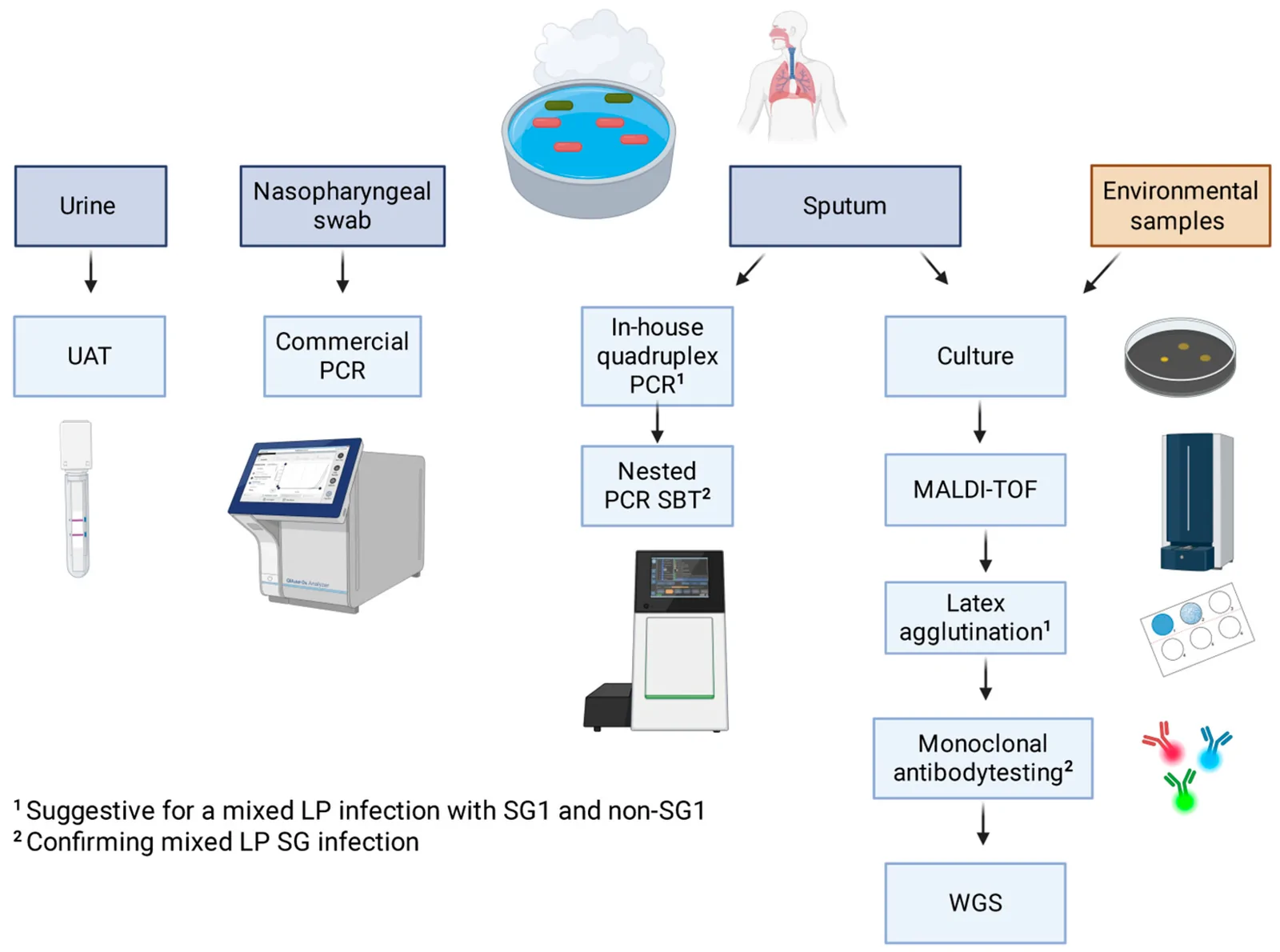
Diagnostic steps used at the NRC Lp to identify Lp SGs in clinical and environmental samples.

SBT directly on sample [23] was applied to the sputum. The obtained partial allelic profile also corresponded to a mixture of both ST1267 and ST146 alleles (see Supplementary Table S2 and Figures S2–S4). Preliminary SG determination using *mip* gene sequence similarity analysis [24] revealed SG10 for the non-SG1 strain (see Supplementary Figure S5). Monoclonal antibody serotyping of Lp isolates by indirect immunofluorescence [25] also confirmed the SG10 result for the non-SG1 strain. The laboratory workflow used for the identification and typing of Lp is illustrated in Figure 2.

Based on clinical, radiological, epidemiological, and molecular evidence, a diagnosis of community-acquired LD due to mixed Lp strains was established.

The patient was admitted for observation and further diagnostic work-up, including blood and sputum cultures, which were obtained shortly after presentation and before initiation of antimicrobial therapy. Given her clinical stability and personal preference, short-term outpatient management with close follow-up was arranged. She was successfully treated with oral levofloxacin (500 mg twice daily) for a total duration of 7 days, according to local guidelines, with clinical improvement observed within 36 h after initiation of therapy. No treatment-related adverse events were reported, and she showed good clinical recovery at follow-up.

## 3. Discussion and Review of the Literature

To our knowledge, the present case represents the first published Belgian case of mixed Lp SG infection and highlights the added value of complementary diagnostic approaches for its detection. Mixed Lp infections, historically considered rare, are likely underestimated due to current limitations in standard routine diagnostics. Recent advances in molecular diagnostics now allow these co-infections to be more readily detected, and they have been documented across various patient populations and clinical settings [4]. As the global incidence of LD continues to rise and the relationship with environmental management remains unclear, accurate diagnosis and typing of *Legionella* isolates involved in clinical cases are essential [13].

An important lesson from this case is that discordant laboratory findings may reveal biological complexity rather than analytical error. The discrepancy between the SG1-specific PCR result and the initial identification of a non-SG1 isolate prompted further investigation, ultimately revealing a mixed Lp SG infection. This finding would have remained unrecognized had diagnosis relied solely on UAT, routine molecular testing, or characterization of a single cultured isolate. Although PCR has substantially improved the sensitivity of *Legionella* detection and is increasingly used as a first-line diagnostic tool, most commercial assays are designed to establish the presence of *Legionella* rather than characterize strain diversity. A mixed infection would not have been seen on the PCR curves. In our case, recognition of the mixed infection was facilitated by the inclusion of an SG1-specific target in the NRC assay, careful interpretation of amplification curves, and subsequent multi-colony analysis. However, in most cases these are not foreseen. Awareness of such mixed infections remains limited, highlighting the need for a diagnostic workflow based on a combination of culture and molecular testing on lower respiratory tract samples to improve detection of non-SG1 or mixed infections.

To contextualize this case, we conducted a narrative literature review to identify published reports of mixed Lp SG infections published between 1980 and 2025. PubMed and Embase were searched until December 2025 using the terms “*Legionella pneumophila*”, “mixed infection” and “serogroup”. Eligible studies included case reports and case series documenting isolation of two or more Lp SGs from the same patient. Extracted data included patient characteristics, diagnostic methods, clinical outcomes, and source identification. Reference lists of included articles were also screened to identify additional relevant publications. A total of 11 studies met the inclusion criteria. Their main characteristics are summarized in Table 1.

The present case is consistent with previous reports demonstrating that mixed Lp SG infections occur in both sporadic cases and outbreak settings worldwide. Similar to previously reported cases, our patient had recognized risk factors for LD, including older age (>50 years) and underlying chronic pulmonary disease [35]. There are, however, no reports of comorbidities associated with a higher risk of mixed infections, as the mixed character would most likely be associated with environmental source exposure rather than human predisposition. Since the first reports in the 1980s, a wide variety of SG combinations have been described. Importantly, many of these infections were identified only after systematic examination of multiple colonies, often in combination with molecular typing approaches such as PFGE, SBT, and cgMLST. Together with the findings from the present case, this suggests that the reported frequency of mixed infections is likely influenced by routine diagnostic practices and may not reflect their true occurrence. Moreover, environmental source attribution was not consistently achieved in previously reported cases, highlighting the complexity of linking heterogeneous clinical isolates to environmental reservoirs [4,5,26,27,28,29,30,31,32,33,34]. Collectively, the available evidence indicates that mixed Lp SG infections represent a diagnostically challenging and epidemiologically important phenomenon that may occur more frequently than currently appreciated, warranting comprehensive microbiological investigation.

The detection of multiple SGs within a single patient has important implications as it complicates diagnostic interpretation. Standard tests such as UAT, which primarily detect Lp SG1, may yield false-negative results in non-SG1 infections or in mixed infections where SG1 is present but in low abundance compared with other SGs [2]. Second, treatment decisions based on single-isolate susceptibility testing may be inadequate if other strains are involved.

Antimicrobial resistance in Lp remains poorly characterized, largely due to the limited implementation of routine antimicrobial susceptibility testing (AST) and the absence of official epidemiological cut-off values. Nevertheless, a limited number of resistance mechanisms and isolates exhibiting elevated minimum inhibitory concentrations (MICs) have been reported in two previous studies [36]. In the present study, AST was not performed. However, the co-occurrence of multiple subtypes within a single sample may potentially mask resistance in individual subpopulations. This possibility should be investigated in future studies by performing AST on all detected subtypes.

Even when culture is performed, a single representative colony is typically selected for further analysis [2,30]. This approach risks overlooking the presence of multiple strains within the same clinical or environmental sample. To capture the true diversity of circulating *Legionella* populations, it is essential to systematically examine multiple colonies. Although there are no official recommendations regarding the number of colonies to analyze, Wewalka et al. recommended examining at least five colonies, particularly those with different morphologies [4]. Applying this strategy to clinical and environmental isolates can substantially improve diagnostic accuracy and enhance the reliability of source attribution during outbreak investigations. However, because *Legionella* is a fastidious pathogen, cultures may grow only a single isolate or fail to recover any isolate, despite the presence of multiple strains in the original sample [37]. Molecular methods, such as SBT performed directly on the sample, can therefore provide important complementary evidence of mixed infection that remains hidden in culture-based approaches [5,38].

To further strengthen laboratory capacity, external quality assessment schemes could include control samples containing two or more serotypes, allowing laboratories to test and optimize their workflows for the detection of mixed Lp SG infections. The integration of these tools in routine surveillance and outbreak investigations could significantly improve case detection and control strategies. However, the increasing reliance on UAT and commercial PCR assays has contributed to a decline in culture-based diagnostics in many laboratories. A survey by the College of American Pathologists reported that up to two-thirds of microbiology laboratories in the United States are no longer able to culture Lp isolates [39]. Where such analyses are not available locally, referral to NRCs is recommended for comprehensive testing.

From an epidemiological perspective, outbreak investigations that rely on matching environmental and clinical isolates may misidentify or fail to identify the source or the associated cases if only one strain is detected per patient. When patients harbor multiple SGs or STs, a perfect match with environmental isolates may not be observed. This strain heterogeneity can obscure transmission pathways and may partly explain the frequent lack of concordance between clinical and environmental isolates in outbreak settings. In this case, the travel-associated exposure was assessed within the European Legionnaires’ Disease Surveillance Network/Travel-Associated Legionnaires’ Disease (ELDSNet/TALD). No additional cases were identified, and no environmental sampling was performed, and therefore no source attribution could be established.

Because water systems can harbor multiple Lp strains simultaneously, environmental investigations routinely include sampling from multiple locations to reduce the risk of missing less abundant strains and to support accurate source attribution [40].

In summary, improved laboratory workflows and clinical awareness are required to avoid underrecognition of mixed infections. The involvement of the NRC in all LD cases is essential to facilitate a broader implementation of molecular diagnostics, complemented by culture and targeted typing, which are essential for accurate diagnosis, optimal patient care, and effective outbreak control.

## 4. Conclusions

Mixed infections involving multiple Lp SGs are diagnostically challenging and epidemiologically relevant but remain underrecognized. Their detection requires comprehensive diagnostic approaches combining culture with analysis of multiple colonies and molecular diagnostics, as routine strategies based on urinary antigen testing, commercial molecular assays, or characterization of a single isolate may fail to capture within-host strain diversity. This case highlights again the limitations of UAT and the added value of integrating advanced molecular methods into NRC workflows. Increased awareness among clinicians, microbiologists, and public health authorities is important to improve recognition of mixed infections and to optimize patient management and outbreak response.

## Figures and Tables

**Table 1:** Reported cases of mixed Lp SG infections in the literature (1980–2025).

Year	Author	Country	Patient Characteristics	Serogroups	Diagnostic Methods	Outcome	Source Investigation	References
1980	Meyer et al.	USA	1 patient with comorbidities, nosocomial infection	SG1, SG4	Culture, DFA	Died	No	[26]
1988	Horbach et al.	Germany	3 patients, no patient characteristics reported	SG1, SG4, SG10	Culture, DFA, FTIR	Not reported	No	[27]
1990	Aubert et al.	France	1 neonate, nosocomial infection	SG1, SG8	Culture	Survived	Yes (hospital)	[28]
1997	Jalaludin et al.	Australia	74-year-old male, no immunosuppression	SG1, SG5	Culture, serology	Died	No	[29]
1998	Lück et al.	Germany	1 postoperative patient, nosocomial infection	SG1, SG12	Culture, PFGE, MAb	Survived	Yes (hospital)	[30]
2013	Yiallouros et al.	Cyprus	9 neonates in a nosocomial outbreak (mixed infection detected in 1 patient postmortem).	SG1, SG3	Culture, DFA, MAB, UAT, serology	Died	Yes (hospital)	[31]
2014	Wewalka et al.	Austria, Denmark, Germany, UK	5 patients, mostly immunocompromised	Various SGs (SG1, SG3, SG6, SG11)	Culture, PCR, SBT	2/5 died	3/5 confirmed (hospital)	[4]
2014	Coscollá et al.	Spain	41 outbreak patients (mixed infection detected in 3 patients)	Not reported. Different STs (ST1393, ST1394)	Culture, PCR, SBT	Survived	No	[5]
2014	Zhang et al.	China	77-year-old with comorbidities	SG5, SG10	Culture, PCR, SBT, PFGE	Survived	No	[32]
2017	Kuroki et al.	Japan	7 male outbreak patients, several with comorbidities (diabetes, liver disease). Mixed infection detected in 1 patient.	SG1, SG13	Culture, SBT, UAT, PFGE, cgMLST	Survived	Yes (spa)	[33]
2025	Najeeb et al.	Canada	1 patient, nosocomial infection	SG1, non-SG1	Culture, cgMLST	Not reported	Yes (hospital)	[34]

Abbreviations: DFA, direct fluorescent antibody; FTIR, Fourier transform infrared spectroscopy; MAb, monoclonal antibody typing; PCR, polymerase chain reaction; SBT, sequence-based typing; PFGE, pulsed-field gel electrophoresis; cgMLST, core genome multilocus sequence typing; ST, sequence type.

## Data Availability

All data generated or analyzed during this study are included in this published article and its Supplementary Information Files.

## Supplementary Materials

The following supporting information can be downloaded at: https://www.mdpi.com/article/10.3390/idr18050093/s1, Figure S1: Real-time polymerase chain reaction (PCR) amplification curve of the *Legionella pneumophila*-specific target generated by the QIAstat-Dx Respiratory SARS-CoV-2 Panel. Fluorescence is plotted against PCR cycle number, Figure S2: SBT results obtained directly from the respiratory sample. Exact allele matches were obtained for six of the seven SBT loci (*flaA*, *pilE*, *asd*, *mip*, *mompS*, and *proA*). No exact match was obtained for *neuA*, Figure S3: Whole-genome sequencing (WGS)-derived SBT results and locus coverage metrics for the Lp SG 10 isolate (ST1267), Figure S4: Whole-genome sequencing (WGS)-derived SBT results and locus coverage metrics for the Lp SG 1 isolate (ST146), Figure S5: *Mip*-based sequence similarity analysis of the non-SG1 Lp isolate; Table S1: Trends in incidence and diagnostic practices of *Legionella pneumophila* infections in the EU/EEA based on ECDC surveillance data, 2015–2023, Table S2: Allelic profiles of *Legionella pneumophila* strains in a mixed infection, comparing direct sequence-based typing (SBT) from the respiratory sample with SBT of individual isolates (SG1 and SG10). Alleles highlighted in green correspond to alleles detected by direct SBT of the respiratory sample.

## Abbreviations

The following abbreviations are used in this manuscript:

AST	Antimicrobial susceptibility testing
BCYE	Buffered charcoal yeast extract
BMPA	BCYE + polymyxin B, anisomycin, cefamandole
cgMLST	Core genome multilocus sequence typing
CT	Computed tomography
Cq	Quantification cycle
DFA	Direct fluorescent antibody
ECDC	European Centre for Disease Prevention and Control
ELDSNet	European Legionnaires’ Disease Surveillance Network
ESGLI	European Society of Clinical Microbiology and Infectious Diseases Study Group for *Legionella* Infections
EU/EEA	European Union/European Economic Area
FTIR	Fourier transform infrared spectroscopy
IFA	Indirect immunofluorescence assay
LD	Legionnaires’ disease
Lp	*Legionella pneumophila*
MAb	Monoclonal antibody
MALDI-TOF	Matrix-assisted laser desorption/ionization time-of-flight
MIC	Minimum inhibitory concentrations
PCR	Polymerase chain reaction
PFGE	Pulsed-field gel electrophoresis
SG	Serogroup
SBT	Sequence-based typing
ST	Sequence type
TALD	Travel-Associated Legionnaires’ Disease
UAT	Urinary antigen test
WGS	Whole genome sequencing
NRC	National Reference Centre
