# Supplementary material for: Mixed Legionella pneumophila Serogroup Infection in a Single Patient: Case Report and Literature Review
Source: Infect Dis Rep. 2026 Aug 26;18(5):93. doi: 10.3390/idr18050093 (PMC13600221; doi:10.3390/idr18050093)
Supplement: Supplementary file 1 [file idr-18-00093-s001.zip › idr-4507010-supplementary.pdf]

Supplementary Materials

Supplementary Table S1: Trends in incidence and diagnostic practices of *Legionella pneumophila* infections in the EU/EEA based on ECDC surveillance data, 2015–2023

Year	Confirmed cases Lp (n)	Notification rate (per 100 000 inhabitants)	UAT (%)	Culture (%)	Lp mixed SG (n)
2015	6 573	1.4	NR	NR	NR
2016	6 560	1.4	NR	NR	NR
2017	8 624	1.8	NR	NR	NR
2018	10 672	2.2	NR	NR	1
2019	10 636	2.2	90%	10%	3
2020	7 712	1.9	87%	11%	3
2021	10 004	2.4	89%	11%	3
2022	11 447	2.6	81%	10%	0
2023	13 493	3.2	79%	10%	3

Abbreviations: EU/EEA, European Union/European Economic Area; *Lp*, *Legionella pneumophila*; UAT, urinary antigen test; NR, not reported.

Supplementary Table S2: Allelic profiles of *Legionella pneumophila* strains in a mixed infection, comparing direct sequence-based typing (SBT) from the respiratory sample with SBT of individual isolates (SG1 and SG10). Alleles highlighted in green correspond to alleles detected by direct SBT of the respiratory sample.

Allele locus	Direct SBT sample	SBT SG1 isolate	SBT SG10 isolate
flaA	2	2	2
pilE	6	10	6
asd	48	18	48
mip	6	10	6
mompS	48	2	48
proA	1	1	5
neuA	No exact match	6	40
**Sequence type (ST)**	Not assignable	146	1267

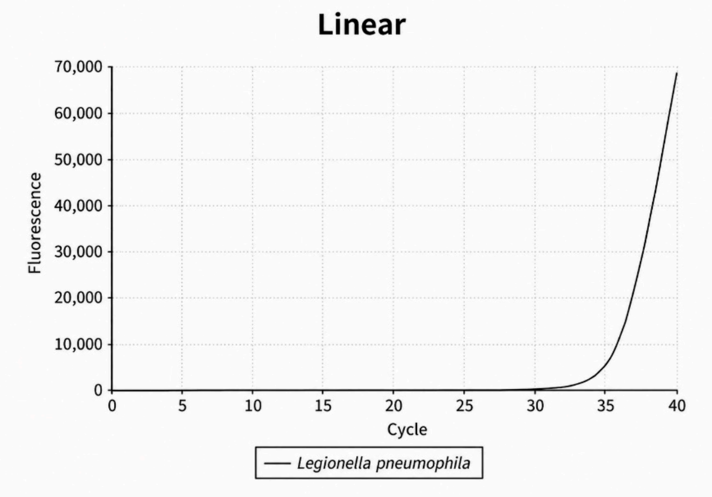

Detected **Legionella pneumophila**

User roel

Test Status Completed
Internal Controls Passed

RESULT DETAILS				Ct / EP
**Viruses**		Not detected	Adenovirus	– / –
		Not detected	Bocavirus	– / –
		Not detected	Coronavirus 229E	– / –
		Not detected	Coronavirus HKU1	– / –
		Not detected	Coronavirus NL63	– / –
		Not detected	Coronavirus OC43	– / –
		Not detected	Human Metapneumovirus A+B	– / –
		Not detected	Influenza A	– / –
		Not detected	Influenza A H1	– / –
		Not detected	Influenza A H1N1 pdm09	– / –
		Not detected	Influenza A H3	– / –
		Not detected	Influenza B	– / –
		Not detected	Parainfluenza virus 1	– / –
		Not detected	Parainfluenza virus 2	– / –
		Not detected	Parainfluenza virus 3	– / –
		Not detected	Parainfluenza virus 4	– / –
		Not detected	Respiratory Syncytial Virus A+B	– / –
		Not detected	Rhinovirus/Enterovirus	– / –
		Not detected	SARS-CoV-2	– / –
**Bacteria**		Not detected	*Bordetella pertussis*	– / –
		Not detected	*Chlamydophila pneumoniae*	– / –
	●	Detected	*Legionella pneumophila*	35.5 / 67,855
		Not detected	*Mycoplasma pneumoniae*	– / –
**Controls**	●	Detected	IC	37.0 / 47,602

Supplementary Figure S1: Real-time polymerase chain reaction (PCR) amplification curve of the *Legionella pneumophila*-specific target generated by the QIAstat-Dx Respiratory SARS-CoV-2 Panel. Fluorescence is plotted against PCR cycle number.

Allele	Length	Average Quality	Start Marker?	End Marker?	Correct Size	Sequence ID's(Length) (Average Quality)	Closest Match	Exact Match	Quality Test Pass
flaA	498	82.79	yes	yes	yes	0319002_flaA+M13uni-21.ab1(324)(56.73)0319002_flaA+M13rev-29.ab1(471)(59.21)	0	2	yes
pilE	502	87.54	yes	yes	yes	0319002_pilE+M13uni-21.ab1(437)(66.00)0319002_pilE+M13rev-29.ab1(479)(62.83)	0	6	yes
asd	640	87.56	yes	yes	yes	0319002_asd+M13uni-21.ab1(555)(65.81)0319002_asd+M13rev-29.ab1(607)(62.83)	0	48	yes
mip	569	90.00	yes	yes	yes	0319002_mip+M13uni-21.ab1(542)(65.89)0319002_mip+M13rev-29.ab1(537)(65.62)	0	6	yes
mompS	553	90.00	yes	yes	yes	0319002_mompS+M13uni-21.ab1(515)(65.75)0319002_mompS+M13rev-29.ab1(521)(66.45)	0	48	yes
proA	493	89.60	yes	yes	yes	0319002_proA+M13uni-21.ab1(463)(65.31)0319002_proA+M13rev-29.ab1(459)(64.66)	0	1	yes
neuA	529	83.28	yes	yes	yes	0319002_neuA+M13uni-21.ab1(436)(59.12)0319002_neuA+M13rev-29.ab1(504)(57.77)	50	0	yes

Sequence Type match = ST none

Supplementary Figure S2: SBT results obtained directly from the respiratory sample. Exact allele matches were obtained for six of the seven SBT loci (*flaA*, *pilE*, *asd*, *mip*, *mompS*, and *proA*). No exact match was obtained for *neuA*.

Sample ID	ST	flaA	pilE	asd	mip	mompS	proA	neuA
Microbiology-LEG1453-MGI-Fast_S20_L01	1267	2	6	48	6	48	5	40

Locus Information

Locus	Percent Covered	Mean Depth	Minimum Depth	Low depth bases
flaA	100.0	3836.9	3163	0
pilE	100.0	4145.6	3652	0
asd	100.0	4325.3	3786	0
mip	100.0	3633.7	3264	0
mompS	100.0	9891.7	8320	0
proA	100.0	4615.7	4000	0
neuA	100.0	4247.7	3823	0

Supplementary Figure S3: Whole-genome sequencing (WGS)-derived SBT results and locus coverage metrics for the Lp SG 10 isolate (ST1267).

Sample ID	ST	flaA	pilE	asd	mip	mompS	proA	neuA
Microbiology-LEG1455-MG I-Fast_S26_L01	146	2	10	18	10	2	1	6

Locus Information

Locus	Percent Covered	Mean Depth	Minimum Depth	Low depth bases
flaA	100.0	3286.5	2712	0
pilE	100.0	3165.3	2806	0
asd	100.0	3541.0	2980	0
mip	100.0	3090.4	2735	0
mompS	100.0	7352.5	6410	0
proA	100.0	3633.8	3068	0
neuA	100.0	3386.9	2873	0

Supplementary Figure S4: Whole-genome sequencing (WGS)-derived SBT results and locus coverage metrics for the Lp SG 1 isolate (ST146).

Because your submitted sequence was a trace file, it was assesed for quality.
Length before trimming: **570** Trimming OK
Length after trimming (3 bases of phred quality score 10 or less allowed in any 25bp region, minimum length after trimming 300): **541**

Species	Percent id
**L.pneumophila_97-2898**	99.26
**L.pneumophila_sg10**	99.08
**L.pneumophila_D-15**	98.89
**L.pneumophila_Wads**	98.89
**L.pneumophila_sg_9**	98.89
**L.pneumophila_Phil-1**	97.97
**L.moravica**	81.70
**L.shakespearei**	80.22

We suggest that to have confidence in the identification the top 'hit' should match with at least 95% identity and the second closest species match should match a value of at least 2% less than the top 'hit'.

Supplementary Figure S5: *Mip*-based sequence similarity analysis of the non-SG1 LP isolate.

Supplementary Methods

The National Reference Centre (NRC) Lp receives samples and isolates on a voluntary basis for diagnostic confirmation and epidemiological surveillance of Legionnaires' disease (LD) cases in Belgium. All respiratory samples are initially tested using an in-house PCR assay, after which PCR-positive samples are further cultured. The culture-positive isolates are subsequently typed by SBT, as described below.

Culture

The respiratory sample was cultured on buffered charcoal yeast extract (BCYE) agar and BCYE supplemented with polymyxin B, anisomycin, and cefamandole (BMPA) (Oxoid, UK), and incubated at 35°C in a humidified atmosphere with 5% CO_2 for up to 10 days. Colonies suspected of belonging to *Legionella spp*. were identified using matrix-assisted laser desorption/ionization time-of-flight (MALDI-TOF) mass spectrometry (Bruker Daltonics, Germany).

Serotyping

Initial serogrouping of *L. pneumophila* isolates was performed using the DrySpot Legionella Latex Agglutination Test (Oxoid, UK), allowing differentiation between serogroup 1 and serogroups 2–15. Isolates classified as serogroups 2–15 were further characterized by monoclonal antibody subgrouping combined with indirect immunofluorescence (IFA), as previously described [25].

Sequence-based typing of clinical isolates

SBT on isolates was performed using whole-genome sequencing (WGS), as previously described [22]. Sequence types (STs) were assigned from the allelic profiles of the seven standard SBT loci (*flaA*, *pilE*, *asd*, *mip*, *mompS*, *proA*, and *neuA*), extracted from WGS data and analysed using the ESGLI SBT database [18, 19]. Bioinformatic analyses were conducted using the MOST and El Gato pipelines [20, 21].

Direct sequence-based typing of respiratory samples

Direct SBT on respiratory samples was performed using the validated ESGLI nested-PCR protocol. DNA extracted from respiratory specimens was subjected to nested PCR amplification of the seven standard SBT loci (*flaA*, *pilE*, *asd*, *mip*, *mompS*, *proA*, and *neuA*), followed by bidirectional Sanger sequencing. Allele numbers and STs were assigned using the ESGLI SBT database [23].

Mip sequencing

The *mip* gene sequence obtained by Sanger sequencing was analysed using the UK Health Security Agency (UKHSA) *mip* Sequence Interrogation Tool, which provides species identification together with supportive information for presumptive serogroup assignment based on sequence similarity [24]. Although *mip* sequencing provides reliable species-level identification, serogroup assignment based on a single gene target has limitations and was therefore considered presumptive. Final serogroup determination was performed by monoclonal antibody serotyping.
